# Pharmacokinetics of anti-TB drugs in Malawian children: reconsidering the role of ethambutol

**DOI:** 10.1093/jac/dkv039

**Published:** 2015-03-10

**Authors:** R. Mlotha, D. Waterhouse, F. Dzinjalamala, A. Ardrey, E. Molyneux, G. R. Davies, S. Ward

**Affiliations:** 1 Department of Paediatrics, Queen Elizabeth Central Hospital, Blantyre, Malawi; 2 Department of Molecular Parasitology, Liverpool School of Tropical Medicine, Liverpool, UK; 3 Faculty of Pharmacy, College of Medicine, University of Malawi, Blantyre, Malawi; 4 Institutes of Infection and Global Health and Translational Medicine, University of Liverpool, Liverpool, UK

**Keywords:** tuberculosis, paediatrics, PK, Africa

## Abstract

**Background:**

Current guidelines for dosing of anti-TB drugs in children advocate higher doses for rifampicin and isoniazid despite limited availability of paediatric data on the pharmacokinetics of these drugs, especially from Africa, where the burden of childhood disease remains high.

**Methods:**

Thirty children aged 6 months to 15 years underwent intensive pharmacokinetic sampling for first-line anti-TB drugs at Queen Elizabeth Central Hospital, Blantyre, Malawi. Rifampicin, isoniazid, pyrazinamide and ethambutol were dosed at 10, 5, 25 and 20 mg/kg, respectively. Plasma drug concentrations were determined using sensitive, validated bioanalytical methods and summary pharmacokinetic parameters were estimated using non-compartmental analysis.

**Results:**

The median (IQR) *C*_max_ was 2.90 (2.08–3.43), 3.37 (2.55–4.59), 34.60 (32.30–40.90) and 1.20 (0.85–1.68) mg/L while the median (IQR) AUC_0–∞_ was 16.92 (11.10–22.74), 11.48 (7.35–18.93), 333.50 (279.50–487.2) and 8.65 (5.96–11.47) mg·h/L for rifampicin, isoniazid, pyrazinamide and ethambutol, respectively. For all drugs, pharmacokinetic parameters relating to drug absorption and exposure were lower than those published for adults, though similar to existing paediatric data from sub-Saharan Africa. Weight and/or dose predicted at least one measure of exposure for all drugs. Age-related decreases in CL/F for rifampicin and pyrazinamide and a biphasic elimination pattern of isoniazid were observed. Predicted AUC_0__–∞_ for rifampicin dosed at 15 mg/kg was comparable to that of adults while the dose required to achieve ethambutol exposure similar to that in adults was 55 mg/kg or higher.

**Conclusions:**

These data support recently revised WHO recommendations for dosing of anti-TB drugs in children, but dosing of ethambutol in children also appears inadequate by comparison with adult pharmacokinetic data.

## Introduction

Clinical diagnosis and treatment of childhood TB is challenging, particularly in resource-poor settings where the burden of disease is highest.^1^ Consequently much of the evidence supporting treatment of paediatric TB is derived from clinical trials of interventions in adults. Appropriate paediatric investigation plans were not carried out for existing first-line anti-TB drugs^2^ and dosing recommendations have been derived from schedules employed for adults, with a relative lack of supportive data on efficacy or safety.^3–5^ In 2010, on the basis of limited existing pharmacokinetic (PK) studies, the WHO recommended that weight-based doses for rifampicin and isoniazid in children should be increased.^6^ However, there remains a paucity of reliable data on the PK of these drugs in children. The current literature relating to first-line drugs in the treatment of active disease from sub-Saharan Africa comprises data from only 133 South African and 45 Malawian children.^7–12^ Furthermore, most of these studies employed relatively sparse sampling schemes, did not include data for all of the first-line drugs deployed in the regimens studied and presented only descriptive analyses. Only a single small study has subsequently reported data on PK associated with the revised WHO recommendations.^13^ We report the results of a PK study employing intensive sampling at steady-state in children representative of the spectrum of paediatric TB in Blantyre, Malawi, including those with HIV coinfection and malnutrition.

## Methods

### Clinical protocol

Children were recruited from the Department of Paediatrics at Queen Elizabeth Central Hospital, Blantyre from January 2007 to February 2008. All parents or guardians gave written informed consent for study procedures. The study protocol was approved by the Ethics Committee of the College of Medicine of the University of Malawi. Children were eligible if they were aged between 6 months and 15 years and had been commenced on treatment for TB as inpatients or outpatients. Baseline evaluation included TB contact history and Mantoux testing using 0.1 mL of tuberculin PPD RT23 (1: 1000), read between 48 and 72 h. Induration of 10 mm or more (or 6 mm or more in an HIV-infected child) was regarded as positive. Chest radiographs were considered suggestive of pulmonary TB in the presence of mediastinal adenopathy, perihilar adenopathy or persistent lobar consolidation. The diagnosis of tuberculous meningitis was accepted with appropriate CSF changes in addition to clinical signs. Following counselling, HIV status was determined by enzyme-linked immunoassay using HIV DETERMINE (Invernos Med, Japan Co Ltd) and confirmed by a second assay with UNIGOLD (Trinity Biotech PK Ireland). CD4 counts were determined for HIV-infected children. Body mass (kg) and height (m) were measured by standard anthropometric methods. Classification of nutritional state was based on the Waterlow^14^ classification of weight for height: >90% was taken as normal; 81%–90% as mild; 70%–80% as moderate; and <70% as severe wasting. Haematocrits were assessed and children with values <25% were not enrolled. Other laboratory tests performed included tests for serum alanine transferase and creatinine.

All the children were recruited at least 2 weeks after initiation of the intensive phase of treatment and received fixed-dose combinations (FDCs) of anti-TB drugs approved by the National TB Programme. Each FDC tablet or sachet contained 60 mg of rifampicin, 30 mg of isoniazid and 150 mg of pyrazinamide, supplemented by loose 100 mg tablets of ethambutol. Doses were administered orally once daily within the following weight bands: <7 kg, 1 tablet; 8–9 kg, 1.5 tablets; 10–14 kg, 2 tablets; 15–19 kg, 3 tablets; 20–24 kg, 4 tablets; and 25–29 kg, 5 tablets. This schedule corresponds to the weight-based doses recommended by the national programme at the time of the study: isoniazid, 5 mg/kg; rifampicin, 10 mg/kg; pyrazinamide, 25 mg/kg; and ethambutol, 20 mg/kg. Children with tuberculous meningitis received intramuscular streptomycin instead of ethambutol.

PK sampling was performed at the hospital at least 2 weeks after treatment initiation. Dosing was administered under observation by ward nursing staff with no restrictions on access to food and water. An intravenous cannula was inserted and maintained using heparin–saline flushes. Blood samples were collected at 0, 0.5, 1, 2, 3, 4, 6, 8 and 24 h into lithium heparin tubes, centrifuged immediately and the separated plasma frozen at −70°C until bioanalysis was performed.

### Bioanalytical methods

Rifampicin and pyrazinamide plasma concentrations were determined using HPLC on a Shimadzu LC 2010 HT system (Shimadzu, Manchester, UK). Isoniazid and ethambutol concentrations were determined simultaneously using LC-MS/MS on a triple-quadrupole TSQ Quantum Access mass spectrometer (Thermo Scientific, Hemel Hempstead, UK). All methods incorporated appropriate internal standards and were validated to internationally recognized acceptance criteria. The lower limits of quantification for the assays were 0.5, 2.5, 0.020 and 0.010 mg/L for rifampicin, pyrazinamide, isoniazid and ethambutol, respectively. Full details of the bioanalytical methods are contained in the supplementary methods (available as Supplementary data at *JAC* Online).

### Statistical analysis

Non-compartmental PK analysis of plasma concentration–time data was performed using Kinetica 4.1.1 (Adept Scientific Ltd, Armor Way, Letchworth Garden City, UK) using the trapezoidal rule with the log up–linear down option and manual adjustment of the range of included timepoints. Data summaries, graphics and analysis of variance of the summary PK parameters were performed in R 2.14.1 (R Foundation for Statistical Computing, Vienna, Austria). Analysis of variance was performed with log-transformed PK parameters where appropriate and model assumptions checked using routine graphical diagnostics. Generalized additive models were used to evaluate continuous covariate relationships using the package mgcV and clustering analysis for subpopulation detection in the parameter distributions was performed using the package mclust.

## Results

Table 1 shows the characteristics of children recruited into the study. Thirty children aged between 6 months and 15 years were enrolled with a mean age and weight of 7 years and 18 kg, respectively. Nine children were aged <2 years and 50% were female. The most common form of TB was pulmonary (21 patients, 70% of children), and the rest of the cases were extrapulmonary, including lymph node and meningeal TB. Twenty (67%) were HIV infected. Eighteen of the HIV-infected children (90%) were receiving co-trimoxazole prophylaxis and nine (45%) were on ART at the time of PK sampling.

**Table 1.** Study population (*N* = 30)

Age (months), median (range)	90 (7–187)
Male, *n* (%)	15 (50)
HIV positive, *n* (%)	20 (67)
Weight (kg), median (range)	18 (4.8–45)
Height (m), median (range)	102 (60–150)
Pulmonary TB, *n* (%)	21 (70)
Co-trimoxazole prophylaxis, *n* (%)	18 (60)
ART, *n* (%)	9 (30)

**Figure 1.**
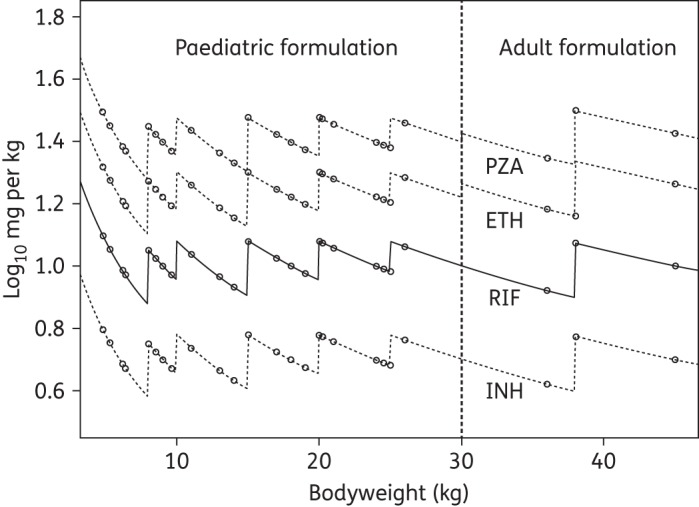
Weight-adjusted dose by weight band for the four drugs in two different formulations. PZA, pyrazinamide; ETH, ethambutol; RIF, rifampicin; INH, isoniazid.

The range of actual weight-adjusted dose for each drug contained in the FDC product (rifampicin, isoniazid and pyrazinamide) and the loose ethambutol tablets is presented in Figure 1. The recommended weight-banding controls the weight-adjusted dose within a narrow proportional range for each drug, although the absolute range of mg/kg dose increased with dose. Hence, the absolute range was largest for pyrazinamide (almost 10 mg/kg across each weight band) and smallest for isoniazid (<2 mg/kg across each weight band). Three children received a similar weight-adjusted dose using the adult formulations of the drugs.

**Figure 2.**
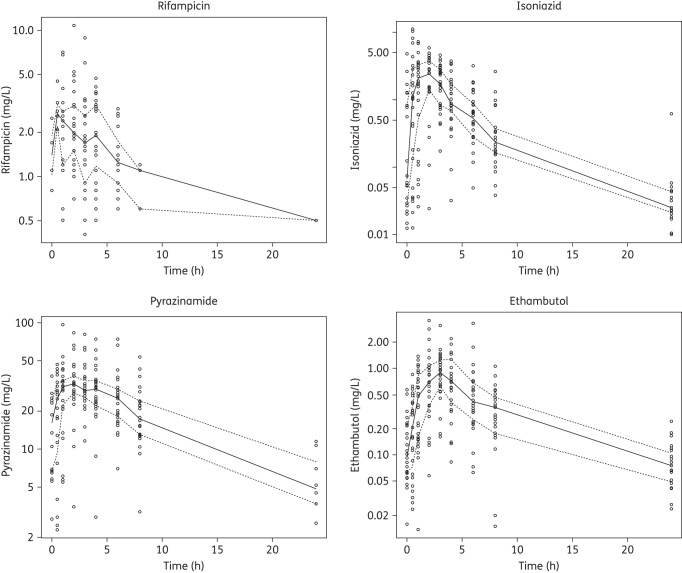
Summary semi-logarithmic scatterplots of plasma concentrations of anti-TB drugs. Continuous lines represent median concentrations and broken lines represent upper and lower quartiles.

Plasma concentrations for each of the drugs over time are summarized in Figure 2. Summary PK parameters derived from non-compartmental analysis are summarized in Table 2. Owing to data below the limit of quantification (LOQ) of the PK assay or a non-credible PK profile, parameters of rifampicin, isoniazid, pyrazinamide and ethambutol could only be estimated for 28, 30, 29 and 28 of the children with mean percentage extrapolation of the AUC_0–∞_ of 28.6%, 3.2%, 30.8% and 10.9%, respectively; 54.4%, 11.1%, 21.1% and 10.3% of datapoints for rifampicin, isoniazid, pyrazinamide and ethambutol, respectively, were below the LOQ and were omitted from the analysis. Estimates of the apparent terminal elimination half-life were based on at least three observations with means of 3.8, 3.6, 3.7 and 3.6 for the four drugs, respectively.

**Table 2.** Summary PK parameters derived from non-compartmental analysis

	Rifampicin (*n* = 28)	Isoniazid (*n* = 30)	Pyrazinamide (*n* = 29)	Ethambutol (*n* = 28)
*C*_max_ (mg/L)	2.90 (2.08–3.43)	3.37 (2.55–4.59)	34.60 (32.30–40.90)	1.20 (0.85–1.68)
*T*_max_ (h)	2.00 (1.00–4.00)	2.00 (1.00–2.75)	2.00 (1.00–3.00)	3.00 (2.00–4.00)
AUC_0–last_ (mg·h/L)	7.50 (5.59–13.06)	11.21 (7.03–18.40)	194.70 (163.40–382.40)	8.00 (4.92–10.07)
AUC_0–∞_ (mg·h/L)	16.92 (11.10–22.74)^a^	11.48 (7.35–18.93)^b^	333.50 (279.50–487.2)	8.65 (5.96–11.47)
*t*_1/2_ (h)	2.01 (1.64–3.27)	3.54 (2.95–4.50)	5.64 (4.47–6.81)	6.49 (5.69–8.04)
CL/F (L/h)	5.41 (3.38–10.64)	5.67 (4.34–8.27)	0.75 (0.48–1.16)	34.0 (19–43)
*V*_z_/F (L)	20.840 (14.850–43.060)	28.93 (21.6–42.9)	6.193 (3.691–9.334)	289 (207–410)
*V*_ss_/F (L)	33.040 (20.10–49.790)	28.3 (19.2–34.8)	7.642 (4.205–10.280)	333 (229–413)

*C*_max_, maximum observed plasma concentration; *T*_max_, time of maximum observed plasma concentration; AUC_0-last_, area under the curve to last observed plasma concentration; AUC_0-∞_, area under the curve extrapolated to infinity; *t*_1/2_, apparent elimination half-life; CL/F, apparent clearance; *V*_z_/F, volume of distribution; *V*_ss_/F, volume of distribution at steady-state.

Data are presented as median (IQR).

^a^Based on 17 subjects.

^b^Based on 28 subjects.

**Figure 3.**
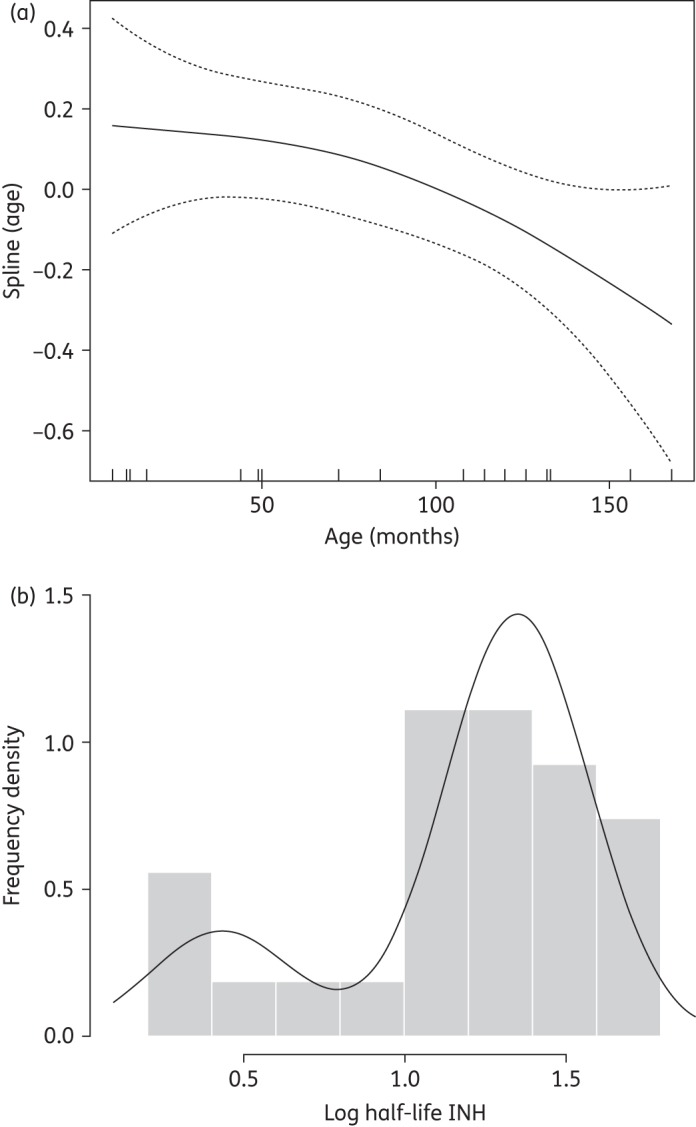
(a) Spline function of rifampicin CL/F with age derived from a generalized additive model (mean estimate shown by the continuous line and 95% Bayesian credible intervals shown by the broken lines). (b) Estimated frequency density of subpopulations of isoniazid CL/F within the dataset derived from empirical clustering analysis. INH, isoniazid.

The mean weight-adjusted dose of rifampicin received was 9.92 mg/kg with a median observed *C*_max_ of 2.90 mg/L and an AUC_0–∞_ of 16.92 mg·h/L (adult reference IQR: 4.2–9.4 and 16.6–36, respectively^14^). Some 87% and 70% of children were below the lower quartile for *C*_max_ and AUC_0–∞_, respectively, in adults. Only 4 of 30 children exceeded the threshold of 4 mg/L cited in adults as representing ‘very low’ exposure to rifampicin. Of note, in eight children absorption appeared to be delayed with a *T*_max_ of 4 h or greater. Mean *C*_max_ in children with delayed absorption was lower than in those with early absorption (2.38 versus 3.58 mg/L), although this was not a statistically significant finding (*t*-test *P* = 0.07). In multivariate analysis, AUC_0–∞_was related to weight-adjusted dose, increasing by 0.12 mg·h/L for each additional mg/kg (*P* = 0.028). This was not true for *C*_max_, possibly because of the delayed absorption observed in some children. CL/F of rifampicin decreased with weight, weight-for-height, age and co-administration of co-trimoxazole in univariate analysis, although only weight remained significant in multivariate analysis. In a generalized additive model CL/F decreased steadily with age after a threshold of ∼4 years, a trend also observed for the highly correlated weight and weight-for-height variables (Figure 3a). Median CL/F for children under 5 years of age was 9.76 L/h, but 4.78 L/h for children over 5 years of age, although this was not a statistically significant difference (Wilcoxon test *P* = 0.41).

The mean weight-adjusted dose of isoniazid was 5.18 mg/kg. The median observed *C*_max_ at 2 h was 3.37 mg/L and the AUC_0–∞_ was 11.48 mg·h/L (adult reference IQR: 4.9–8.7 and 22.5–42.4, respectively^14^). Some 80% and 90% of children were below the lower quartile for *C*_max_ and AUC_0–∞_, respectively, in adults. AUC_0–∞_ did not increase with absolute or weight-adjusted dose (*P* = 0.862 and 0.147). The rate of absorption as represented by *T*_max_ increased with both absolute and weight-adjusted dose (*P* = 0.032 and 0.041). The overall median half-life of isoniazid was 3.54 h, but, as expected, clustering analysis separated the children into two main groups with mean half-lives of 1.54 h (19.9%, likely fast acetylators) and 3.85 h (80.1%, likely intermediate or slow acetylators) (Figure 3b). Of note, 18 of 30 children had detectable concentrations of isoniazid at 24 h post-dose, a finding highly suggestive of biphasic elimination. Re-analysis omitting this timepoint was performed to examine the influence of this information and showed that the overall median half-life would have been estimated at 1.65 h, a figure more similar to many previous reports, with a similar predicted proportion of fast acetylators.

The mean weight-adjusted dose of pyrazinamide was 24.80 mg/kg. The median observed *C*_max_ at 2 h was 34.6 mg/L and the AUC_0–∞_ was 333.50 mg·h/L (adult reference IQR: 46.0–61.4 and 406.2–632.3, respectively^14^). Some 83% and 67% of children were below the lower quartile for *C*_max_ and AUC_0–∞_, respectively, in adults. *C*_max_ was significantly related to absolute dose (*P* = 0.02), but AUC_0–∞_ was not (*P* = 0.11). The *T*_max_ of pyrazinamide increased with absolute dose (*P* = 0.049). The CL/F of pyrazinamide appeared to decrease significantly with age (*P* = 0.036) and this trend appeared linear in a generalized additive model.

The mean weight-adjusted dose of ethambutol was 17.05 mg/kg. The median observed *C*_max_ at 2 h was 1.2 mg/L and the AUC_0–∞_ was 8.65 mg·h/L (adult reference IQR: 4.1–6.3 and 19.2–30.8, respectively^14^). Some 100% and 93% of children were below the lower quartile for *C*_max_ and AUC_0–∞_, respectively, in adults. *C*_max_, AUC_last_ and AUC_0–∞_ were all strongly predicted by absolute, but not weight-adjusted, dose with AUC_0–∞_ increasing by 0.13 mg·h/L for each additional milligram. Age also appeared to affect measures of exposure with AUC_0–∞_ increasing by 0.013 mg·h/L for each additional month of age. Serum creatinine was also a significant predictor for AUC_0–∞_ with exposure increasing by 8.649 mg·h/L for each additional g/dL (*P* = 0.048). Ethambutol exhibited a very high apparent volume of distribution (333 L or 18.5 L/kg).

HIV coinfection was not associated with any significant change in PK parameters.

Co-administration of co-trimoxazole prophylaxis appeared to increase the CL/F of rifampicin alone (*P* = 0.028), while concomitant ART did not impact any PK parameter. Weight-for-height was not identified as an important covariate for any of the drugs or parameters.

Given the complex relationships observed with dose and weight and lack of information on NAT2 genotype, it was only possible to cautiously evaluate alternative dosing recommendations for rifampicin and ethambutol. For rifampicin, assuming a linear relationship with weight-adjusted dose, the expected AUC_0–∞_ at the newly recommended target of 15 mg/kg would be 65.1 mg·h/L, which exceeds the upper quartile of the observed adult range. For ethambutol, however, linear extrapolation of average weight-adjusted dose suggested that an AUC_0–∞_ comparable to adults (>19.2 mg·h/L) could only be achieved at a dose of 55 mg/kg or higher.

## Discussion

This study is the most intensive PK study of all four first-line anti-TB drugs yet undertaken in children with TB and adds substantially to the evidence available, especially in sub-Saharan Africa, which bears the highest burden of childhood disease. All studies of African children have to date been performed in South Africa,^7–11^ with the exception of a single study from Malawi, which evaluated intermittent dosing of pyrazinamide and ethambutol alone.^12^ The size of the cohort, nine-point sampling strategy and sensitive bioanalytical methods adopted in the current study allowed for more accurate and precise estimates of PK parameters using non-compartmental techniques than was previously possible, particularly *C*_max_, which may be underestimated by sparse sampling. More importantly, improved estimates of AUC_0–∞_, believed to be the key determinant of clinical efficacy for most first-line drugs, could also be obtained.

The most important measures of absorption and exposure (*C*_max_ and AUC_0–∞_) for all of the drugs assayed in this representative group of African children with TB were lower than typically observed in African adults using the same analytical approach.^15^ For most drugs, some relationship of exposure with absolute or less commonly weight-adjusted dose was observed for at least one measure of exposure. In addition, for rifampicin and pyrazinamide, there appeared to be an independent relationship of CL/F with age. Isoniazid elimination appeared biphasic, with a longer apparent half-life than previously reported, but a frequency of fast acetylator phenotype similar to reports from South Africa. This finding probably resulted from the availability of a sample at 24 h and the highly sensitive bioanalytical method used for isoniazid. Although observed in some previous studies, the possible metabolic basis of this finding is currently unknown. Exposure of ethambutol appeared to be significantly related to creatinine. Neither HIV nor nutritional status significantly affected the PK parameters of any of the drugs. Co-administration of ART or co-trimoxazole prophylaxis also did not appear to have any impact with the possible exception of a small increase in rifampicin CL/F associated with co-trimoxazole co-administration.

These findings are generally in agreement with limited data available from elsewhere in Africa and support recent recommendations by the WHO to increase the dose of isoniazid and rifampicin in children with TB to 10 and 15 mg/kg, respectively. Although PK targets for treatment success in TB are not well-defined, ensuring exposure in children at least equivalent to adults is logical given the excellent overall performance of first-line therapy in adults. The data presented here might also suggest that dose modification could be considered for pyrazinamide and ethambutol. However, it was difficult to accurately predict the equivalent doses required due to sometimes complex relationships observed between dose, weight and the PK parameters. This may be attributable to the relatively small sample size and in the case of isoniazid to tight weight-banding in a small dose range and confounding effects of acetylator status. Absolute dose size did appear to independently predict exposure of pyrazinamide and ethambutol, however. In the case of ethambutol, extrapolation of this relationship with AUC_0–∞_ appeared to confirm previous suggestions that current dosing of ethambutol in children may be inadequate and that a substantial dose increase would be needed to achieve plasma concentrations comparable to those in adults. This finding is consistent with the results of a previous study in Blantyre, which evaluated thrice weekly dosing at 35 mg/kg, achieving a *C*_max_ of only 1.8 mg/L. These extrapolations assume linearity of PK at higher doses and better predictions might be obtained using a more sophisticated modelling approach and dose-ranging data. A single small study from South Africa has also suggested that the new dosing recommendations can achieve the targeted exposures desired.^13^

In addition to relationships observed with dose and weight, other features relating to the metabolism of certain drugs were observed. Using empirical clustering, an unequivocally fast acetylator phenotype could be attributed to 20% of the children, consistent with previous studies in South Africa, which reported a homozygous slow acetylator NAT2 genotype in 36%–40% of children.^8,10,13^ However, the African region exhibits the greatest diversity at this locus worldwide^16,17^ and further characterization of the distribution of NAT2 alleles in Malawi would be useful in view of the prolonged half-life of isoniazid that was observed. Both rifampicin and pyrazinamide appeared to exhibit age-related changes in CL/F independent of body size that were judged statistically significant. For pyrazinamide this trend appeared linear over the age range studied, whereas for rifampicin CL/F appeared to decrease around 4 years, ultimately halving over the age range. These changes may reflect the greater relative liver size and blood flow per kilogram in younger children.^18^ Why the shape of these trends should differ is unclear, although it might reflect maturation processes specific to the dominant metabolic pathways for the two drugs (hepatic carboxyesterases for rifampicin and amidase for pyrazinamide). The apparent CL/F of pyrazinamide reported here is lower than that observed in adults, but this could reflect changes in bioavailability relative to adults rather than truly reduced clearance, given the sachet formulation used in this study. Ethambutol exposure was related to serum creatinine, which is consistent with the renal route of elimination of the drug. CL/F was not clearly related to serum creatinine, but this may be due to the restricted range of the covariate in this dataset.

From a clinical perspective the study provides insight into dosing in potentially important subgroups and with other commonly co-administered medications. We found no evidence that HIV or nutritional status significantly affected PK parameters or that there are any important PK interactions with co-trimoxazole prophylaxis or nevirapine-based ART. Although the analysis suggested a possible increase in CL/F of rifampicin with co-administration of co-trimoxazole prophylaxis, this has not been reported elsewhere and could be a chance finding requiring independent confirmation. Overall, these data provide reassurance that dosage adjustment of anti-TB drugs is not needed for any of these specific reasons. However, it should be noted that the study was not designed to evaluate different phenotypes of malnutrition and further work would be necessary to substantiate this conclusion.

In conclusion, this intensive PK study demonstrates that plasma exposures of first-line anti-TB drugs in Malawian children are lower than those observed in African adults, confirming the limited data available to date from paediatric PK studies in the region. These findings are supportive of recent WHO recommendations to increase the doses of rifampicin and isoniazid in routine paediatric practice, but the low exposures of ethambutol observed also suggest that the dose and role of this drug in childhood TB should be revisited.

## Funding

This work was supported by the Wellcome Trust UK.

## Transparency declarations

None to declare.

## Supplementary data

Supplementary methods are available as Supplementary data at *JAC* Online (http://jac.oxfordjournals.org/).

Supplementary Data
