# Supplementary material for: Pharmacokinetics of anti-TB drugs in Malawian children: reconsidering the role of ethambutol
Source: J Antimicrob Chemother. 2015 Mar 10;70(6):1798–803. doi: 10.1093/jac/dkv039 (PMC4498297; doi:10.1093/jac/dkv039)
Supplement: Supplementary Data [file supp_70_6_1798__index.html]

Pharmacokinetics of anti-TB drugs in Malawian children: reconsidering the role of ethambutol — Supplementary Data 

# Pharmacokinetics of anti-TB drugs in Malawian children: reconsidering the role of ethambutol

## Supplementary Data

Supplementary Data

**Files in this Data Supplement:**

- Supplementary Data - Doc file
