# Supplementary material for: Pharmacokinetics of anti-TB drugs in Malawian children: reconsidering the role of ethambutol
Source: J Antimicrob Chemother. 2015 Mar 10;70(6):1798–803. doi: 10.1093/jac/dkv039 (PMC4498297; doi:10.1093/jac/dkv039)
Supplement: Supplementary Data [file supp_dkv039_dkv039supp.doc]

**Supplementary data**

Bioanalytical methods

HPLC method for the determination of rifampicin

All sample preparations were carried out in a darkened room due to the light sensitive nature of RIF. Two hundred microliters of each sample were assayed alongside a plasma calibration curve (range 0 - 32 mg/L) with quality control samples at low 1.5 mg/L, medium 17 mg/L, and high 26 mg/L concentrations. Fifty microliters of internal standard (ethanol containing 16 mg/L butyl 4-hydroxybenzoate) was added to all plasma aliquots prior to protein precipitation with 1 mL of methanol. Plasma aliquots were then vortexed for 20 s and left to stand at 4^o^C for 1 h before being centrifuged at 2,000 x g for 10 min. The resultant supernatants were then decanted into a clean 5 mL soda glass tubes and evaporated to dryness under a stream of nitrogen at 30^o^C. The dried down samples were then reconstituted in 120 µL of methanol and vortexed for 10 seconds. From the 120 µL of the reconstitute, a 100 µL aliquot was then subjected to chromatographic separation on a Luna C8 150 x 4.6 mm, 5 µm particle size, column (Phenomenex Inc, Macclesfield, UK) protected by a LiChrospher Si 60 5µm guard column (VWR, Lutterworth, UK) with the column oven set to 30^o^C. Chromatographic separation was achieved using a mobile phase gradient program at a flow rate of 1 mL/min. The initial conditions consisted of 35% solvent A (acetonitrile) and 65% solvent B (50 mM ammonium formate [pH 5], adjusted with formic acid) for initial 0 – 2 min of the run. Solvent A was then increased to 70% from 2-7 min and held there for 1 min. The column was then equilibrated to the initial start conditions over a total run time of 12 min. Samples were analysis using a Shimadzu LC 2010 HT HPLC system (Shimadzu, Manchester, UK), with detection at 254 nm. All standard curves were adequately described using an equal weighted linear regression equation, using data acquisition software Chromeleon (Version 6.7, Dionex, UK). The correlation coefficient (*r^2^*) for all RIF calibration curves exceeded 0.99. The lower limit of quantification (LLOQ, 0.5 mg/L) was accepted as the lowest point on the standard curve, with a signal-to-noise ratio of 5:1 and a coefficient of variation (CV) of less than 10% and ranged between 2% CV and 13% CV at all other calibration levels. The determination of RIF stability following three freeze-thaw cycles showed that all quality control samples, there was a 9% CV in RIF concentrations. RIF stability after heat inactivation showed a 11% CV in RIF concentration.

HPLC method for the determination of pyrazinamide

One hundred microliters of each sample were assayed alongside a plasma calibration curve (range 0 - 80 mg/L) with quality control samples at low 8 mg/L, a medium 38 mg/L, and high 64 mg/L concentrations. All plasma aliquots underwent protein precipitation with 200 µL of internal standard (acetonitrile containing 10 µg/mL acetazolamide). Plasma aliquots were then vortexed for 10 s before being centrifuged at 17,000 x g for 10 min. Two hundred and fifty microliters of the resultant supernatants were then decanted into a clean 5 mL soda glass tubes and evaporated to dryness under a stream of nitrogen at 30^o^C. The dried down samples were then reconstituted in 200 µL of mobile phase consisting of 95% water (0.06% TFA) and 5% acetonitrile and then vortexed for 10 seconds. From the 200 µL of the reconstitute, a 60 µL aliquot was then subjected to chromatographic separation on a HyPURITY C18 (250 x 4.6 mm, 5 µm particle size) column (Thermo Scientific, UK) protected by a LiChrospher Si 100 RP-18 (5µm) guard column (VWR, Lutterworth, UK) with the column oven set to 30^o^C. Chromatographic separation was achieved using an isocratic gradient of 95% water (0.06% TFA) and 5% acetonitrile at a 1 mL/min flow rate. Samples were analysis using a Shimadzu LC 2010 HT HPLC system (Shimadzu, Manchester, UK), with detection at 268 nm. All standard curves were adequately described using an equal weighted linear regression equation, using data acquisition software Chromeleon (Version 6.7, Dionex, UK). The correlation coefficient (*r^2^*) for all PZA calibration curves exceeded 0.99. The lower limit of quantification (LLOQ, 2.5 mg/L) was accepted as the lowest point on the standard curve, with a signal-to-noise ratio of 5:1 and a coefficient of variation (CV) of less than 14% and ranged between 1% CV and 14% CV at all other calibration levels. The determination of PZA stability following three freeze-thaw cycles showed that all quality control samples, there was a 14% CV in PZA concentrations. PZA stability after heat inactivation showed a 14% CV in PZA concentration.

LC-MS/MS method for the determination of isoniazid and ethambutol concentrations

One hundred microliters of each sample were assayed alongside a plasma calibration curve (range 0 – 5 mg/L) with quality control samples at low 0.060 mg/L, medium 2 mg/L, and high 4 mg/L concentrations for isoniazid (INH) and ethambutol (EMB) respectively. All plasma aliquots underwent protein precipitation with 400 µL of internal standard (methanol containing 200 ng/mL metformin). Plasma aliquots were then vortexed for 20 s before being centrifuged at 2,000 x g for 5 min. The resultant supernatants were then decanted into a 10 mL glass test-tube containing 200 µL of water and 2 mL of dichloromethane. This was then vortexed for 1 minute s before being centrifuged at 2,000 x g for 5 min. A 100 µL aliquot of the resultant supernatant was then transferred to a clean 5 mL soda glass tubes and evaporated to dryness under a stream of nitrogen at 45^o^C. The dried down samples were then reconstituted in 200 µL of mobile phase consisting of 90% water, 10% methanol and 0.3%formic acid and then vortexed for 10 seconds. From the 200 µL of the reconstitute, a 10 µL aliquot was then subjected to chromatographic separation on a Hypersil GOLD C18 (150 x 4.6 mm, 3 µm particle size) column (Thermo Scientific, UK) with the column oven set to 30^o^C. Chromatographic separation was achieved using an isocratic gradient of 90% water, 10% methanol and 0.3%formic acid at a 300 µL/min flow rate. The HPLC system was interfaced with a triple-quadrupole TSQ Quantum Access mass spectrometer (Thermo Scientific, Hemel Hempstead, UK) with an atmospheric pressure chemical ionization (APCI) source. An E2M30 rotary vacuum pump (Edwards High Vacuum International, West Sussex, UK), an NM30LA nitrogen generator (Peak Scientific, Renfrewshire, UK) and 99% pure argon gas (10L, BIP10, Air Products, Liverpool, UK) were used.

The triple-quadrupole mass spectrometer was operating in positive selective reaction monitoring (SRM) mode set to a narrow scan width (0.1 *m/z*) and scan time (0.1 s) for all transitions. Data were collected in centroid mode. The sheath and auxiliary gas flow (nitrogen gas) were set to 15 (psi) and 20 (psi) respectively. The capillary temperature within the ion source was maintained at 250^o^C with the discharge current set to 5 µA, the spray voltage set to 4.5 kV with a collision pressure of 1.5 mTorr (Argon). All standard curves were adequately described using an equal weighted linear regression equation for INH and 1/concentration weighted liner regression equation for EMB, using data acquisition software LC Quan (Version 2.5.6.Thermo Scientific, Hemel Hempstead, UK). The correlation coefficient (*r^2^*) for all INH and EMB calibration curves exceeded 0.99. The lower limit of quantification (LLOQ, 0.020 mg/L and 0.010 mg/L for INH and EMB respectively) was accepted as the lowest point on the standard curve, with a signal-to-noise ratio of 5:1 and a coefficient of variation (CV) of less than 11% for both INH and EMB. A ranged between 2% CV and 11% CV at all other calibration levels for both INH and EMB. The determination of INH and EMB stability following three freeze-thaw cycles showed that all quality control samples, where within 11% & 12% CV for INH and EMB concentrations respectively. INH and EMB stability after heat inactivation where within 5% & 8% CV for INH and EMB concentrations respectively.
